# Health problems experienced by women during the first year postpartum: A systematic review

**DOI:** 10.18332/ejm/173417

**Published:** 2023-12-18

**Authors:** Marije M. Gmelig Meyling, M. Evelyn Frieling, Johanna P. M. Vervoort, Esther I. Feijen-de Jong, Danielle E. M. C. Jansen

**Affiliations:** 1 University of Groningen, Groningen, the Netherlands; 2 Department of Health Sciences, University of Groningen, University Medical Center Groningen, Groningen, the Netherlands; 3 Department of Primary and Long-term Care, University of Groningen, University Medical Center Groningen, Groningen, the Netherlands; 4 Department of Midwifery Science, Amsterdam University Medical Centers, Vrije Universiteit Amsterdam, Amsterdam Public Health Research Institute, Amsterdam, Amsterdam, the Netherlands; 5 Midwifery Academy Amsterdam Groningen, Inholland, Groningen, the Netherlands

**Keywords:** maternal health, postpartum period, exhaustion, urinary incontinence, depressive symptoms, systematic review

## Abstract

**INTRODUCTION:**

During pregnancy and childbirth, health issues can arise that can negatively influence women’s postpartum health. Although it is imperative to identify these health problems in order to tailor care to women’s needs, they often remain unrecognized. A comprehensive overview of postpartum health problems does not exist in the current literature. This systematic review aimed to explore the health problems experienced by women residing in high-income countries during the first year postpartum.

**METHODS:**

Scientific databases were searched for articles on health problems experienced by women during the first year postpartum, published between January 2000 and 2 July 2021. Studies investigating the experiences of healthy women from the age of 18 years, residing in high-income countries, who gave birth to a healthy neonate, were included. Identified health issues were divided into five categories and presented in an overview.

**RESULTS:**

A total of 25 articles were eligible for inclusion. In all, 83 health problems were identified and divided into five different categories (physical health problems, mental health problems, social health problems, problems related to feeding the infant, and other challenges). Common health issues postpartum were exhaustion, urinary incontinence, painful breasts, depressive symptoms, problems related to sexuality and sleep, lack of social support, and problems with breastfeeding

**CONCLUSIONS:**

This systematic review contributes to a wider understanding of postpartum health problems and can be used to adapt healthcare to women’s needs. It distinguishes itself from previous studies by the wide variety of identified health problems and its specific focus on women’s experiences in the postpartum period.

## INTRODUCTION

The postpartum period is a period of growth and development for newborns, but also for women as they transition into parenthood^1^. While they feel joy and happiness, women can also experience stress and an overwhelming sense of responsibility^1-3^. Health problems can arise during pregnancy and childbirth, persist during the postpartum period, and can impact women’s physical, psychological, social health, and the health of the newborn^4-12^. The percentage of women in high-income countries experiencing one or more postpartum health problems varies from 47% to 94%^13-15^. Examples include: tiredness^16^, urinary incontinence^17^, depression^18^, and sexual health issues^19-21^.

It is important to recognize health problems experienced by women, as these can relate to women’s healthcare needs. According to the principles of patient-centered care (PCC), healthcare and clinical decisions should be adapted to the needs, preferences, and values of each individual patient^22-24^.This is positively related to satisfaction and wellbeing^24^. Despite these benefits, postpartum consultations mostly seem to focus on the newborn’s wellbeing and not enough attention is paid to the parent’s health problems experienced and needs^1,13,25-27^. This is worrisome, as poor parental health negatively influences parenting behavior and children’s health outcomes^5-9,11^. Additionally, failure to recognize parental health problems could lead to limited satisfaction and wellbeing.

Most studies investigating postpartum health problems focus on objective measurements of health problems and rarely address women’s subjective experiences identified by qualitative research methods^28-31^. A limited number of studies have investigated postpartum health problems in multiple domains, such as the physical, mental, and social health domain; however, these studies were published several years ago and have not been updated^4,10,13,32-35^. A comprehensive up-to-date overview of postpartum health problems experienced by women postpartum, does not exist in current literature.

The aim of this systematic review is to identify health problems that women who have given birth in high-income countries can experience in the first year postpartum, and present these problems in an overview. This review answers the following question: ‘Which physical, mental, and social health problems do women in high-income countries experience in the first year postpartum?’. An updated overview of women’s health problems experienced in the first year postpartum can be used to recognize women’s needs in order to improve short- and long-term health and well-being, and provides information to caregivers for improving health services.

## METHODS

A systematic review was conducted between May 2020 and July 2021. This review was reported according to the Preferred Reporting Items for Systematic Reviews and Meta-analysis (PRISMA) (Supplementary file Section A)^36^. To ensure methodological consistency between authors, a review protocol was developed a priori and registered in PROSPERO (Registration number: CRD42020194123). Interim changes to the protocol were recorded.

### Outcome measures

Primary outcome measures were physical, mental, and social health problems experienced by women in high-income countries during the first year postpartum. Health problems experienced were defined as symptoms, feelings, emotions or perceptions that are considered problematic by the person experiencing them. The original purpose of this systematic review was to provide input for the development of an online course for women from the Netherlands. For this reason, we chose to limit the research question to health problems experienced by women from high-income countries specifically, as women from low-income countries might experience different health problems postpartum due to differences in postpartum healthcare.

### Search strategy and screening process

Search terms and synonyms were used for the following key terms: ‘health problems’, ‘experience’, ‘women’, ‘postpartum period’, and ‘high-income countries’, and were linked with the Boolean operators OR and AND. The search strategy was peer reviewed by all authors and a medical librarian. It was tested and adapted to fit each electronic database (Supplementary file Section B). The search strategy was performed in electronic databases MEDLINE, CINAHL and PsycINFO, on 26 May 2020. The researchers chose to limit the search to these electronic databases, as they were believed to be the most useful data sources to identify articles on the subject of postpartum health problems. A rerun of the search was performed on 2 July 2021. No filters were applied to limit the results.

To be included, studies had to report data from women aged ≥18 years, residing in a high-income country, who had given birth^37^. Studies had to report at least one physical, mental, or social health problem experienced by women during the first year postpartum. Peer-reviewed studies written in English or Dutch, published between January 2000 and 2 July 2021, were included. Studies that explicitly reported on health problems experienced after stillbirth, after giving birth to a premature neonate or a neonate with congenital anomalies were excluded. Furthermore, studies exclusively reporting data from women with preexisting medical conditions, women who used reproductive techniques for conception and women who experienced a high-risk situation during pregnancy or childbirth, were not included in this review because these women may experience different issues postpartum. High-risk situations were defined as all situations classified with code B, C and/or D according to the *Verloskundig Vademecum* 2003^38^. Book reviews, dissertations, conference abstracts, editorials, opinions, and protocols were excluded. Grey literature was not included in this systematic review, as it can be difficult to find and it is often complicated to assess if the information from these sources has been submitted to peer-review and is reliable.

Screening and selection of articles was conducted by two researchers independently. All discrepancies during the review process were discussed until consensus was reached. If necessary, a third person from the review team was consulted. Articles were entered in EndNote X9^39^ and duplicates were removed. Subsequently, all articles were uploaded in Rayyan QCRI^40^ in which remaining duplicates were removed and the screening process was performed. Titles and abstracts were screened and full text was assessed for seemingly eligible articles. Reasons for exclusion were recorded. Reference lists and citations (list of all articles referring to the article in question) were reviewed to identify additional eligible articles not found with the search strategy. These newly identified articles were independently screened for eligibility. Newly published articles, identified in the re-run of the search, were assessed following the same screening process.

### Analysis

Using an a *priori* created data extraction form (Supplementary file Section C), the following data were extracted from included articles: title, author(s), year of publication, country, study design, study population characteristics, outcome measures, postpartum time points of measurement, and relevant results. Characteristics of the included articles were entered into a table and independently verified by two researchers. Only data from women aged ≥18 years in high-income countries and health problems experienced up to 12 months postpartum, were extracted.

The methodological quality of the included articles was independently assessed by two researchers, using study design specific checklists^41-43^. For the quality assessment, a scoring system was used in which each item answered with a ‘yes’ was awarded one point. After converting the total number of points into a percentage using a formula (see review protocol), articles were assigned a low (total score of 0–40%), medium (total score of 41–70%) or high (total score of 71–100%) quality assessment. Low quality articles were excluded from further data synthesis.

An overview of women’s health problems experienced in the first year postpartum was created using data from medium-quality and high-quality articles. Health problems indicated with an umbrella term were not included in this overview, because the definitions of these terms are open to subjective interpretation. For example, ‘breast problems’ may include any problem related to the breasts and is therefore not clearly defined. The identified health problems were divided into categories in accordance with the International Statistical Classification of Diseases and Health Related Problems – tenth revision (ICD-10) and presented in alphabetical order^44^. Health problems described in chapters I – IV and VI – XX of the ICD-10, were classified as ‘Physical health problems’. Health problems described in chapter V: ‘Mental and behavioral disorders’ were classified as ‘Mental health problems’. All health problems described in Chapter XXI: ‘Factors influencing health status and contact with health services’, were classified as ‘Social health problems’.

## RESULTS

### Results of the search strategy

**Figure 1:**
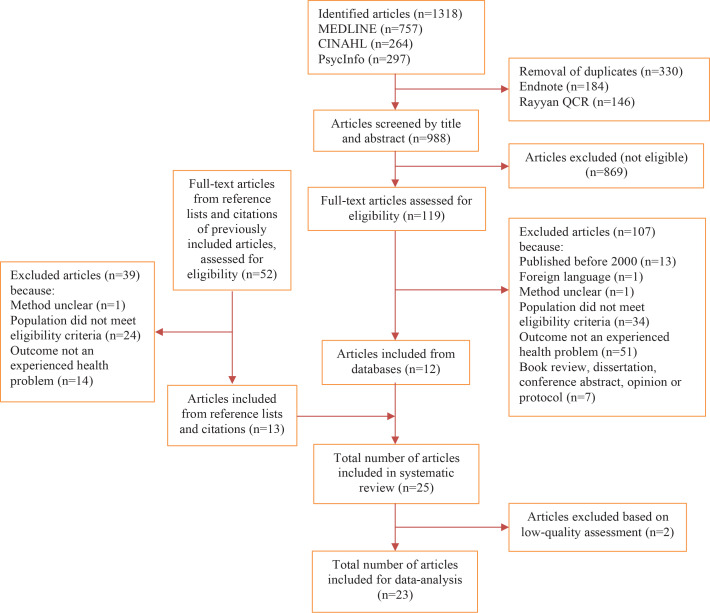
Flowchart summarizing the results of the search strategy

The search resulted in 1318 articles. After removal of duplicates and eligibility assessment of titles and abstracts, 119 full-text articles were assessed on relevance. Of these, 12 articles were eligible for inclusion. The reference lists and citations of these 12 articles were screened for eligibility and 13 additional articles were included. A total of 25 articles were included in this systematic review. The level of agreement between the two researchers after independent screening of the articles was about 98%. The results of the selection process and the reasons for exclusion are shown in Figure 1.

### Characteristics of included studies

Characteristics of the 25 included studies are shown in Table 1. Several included studies were qualitative studies (n=11). In addition, ten prospective cohort studies, two cross-sectional studies, one retrospective study and one mixed-methods study, were included. The studies were conducted in Europe (n=12)^20,25,45-54^, Oceania (n=8)^10,55-61^, North America (n=4)^4,62-64^, and Asia (n=1)^65^. Study populations differed in size between 10 and 4516 women^54,60,63^. Primiparous women were included exclusively in 11 studies, and 14 studies included both primiparous and multiparous women. In most studies (n=15), the majority of women had completed secondary school or were educated to a degree level^4,20,25,46,47,50,52-57,61,63^.

**Table 1:** Characteristics of the included studies

*Authors Year*	*Country*	*Study design and methods*	*Study population*	*Outcome measures*	*Measurement timepoints (postpartum)*	*Relevant results: health problems experienced in the first year postpartum*	*Total score (quality assessment)*
Alianmoghaddam et al.^55^ 2017	New Zealand	Qualitative, longitudinal study Methods: face-to-face interviews with open-ended questions	30 primiparous and multiparous women aged >25 years	Factors influencing the maintenance of exclusive breastfeeding	Between 4–6 weeks Subsequently, monthly until a maximum of 6 months	Women received insufficient advice from their midwives, in the hospital or during pregnancy courses, both before childbirth and in the postpartum period. They felt unprepared for lactation and associated problems. Problems experienced with breastfeeding were: sore nipples, breast pain, perceived insufficient breast milk, difficulty latching on; exhaustion, high societal pressure to breastfeed, and shame about bottle feeding.	80 % (High)
Andrew and Harvey^45^ 2011	England	Qualitative, cross-sectional study Methods: face-to-face interviews with open-ended questions	12 primiparous and multiparous women with a baby aged 7–18 weeks	Factors influencing decisions regarding feeding of the baby Experiences of women with regard to feeding the baby	Between 7–18 weeks	Breastfeeding experiences were: societal pressure to breastfeed, difficulty juggling responsibilities, pain, cracked nipples, difficulty latching the baby onto the nipple, lack of confidence in in milk supply, difficulty finding time to breastfeed, difficulty dividing attention between children, little time for themselves, breastfeeding is time consuming, loss of independence, feelings of embarrassment while breastfeeding in public, and feeling isolated at home.	77.7 % (High)
Ansara et al.^4^ 2005	Canada	Retrospective, quantitative study Methods: telephone interviews with administration of a questionnaire	200 primiparous and multiparous women aged 19–42 years	Presence of common health problems of women in the first year postpartum out of a list of 13 problems	Between 8–10 weeks	96% reported at least one health problem in the first two months postpartum. Health problems experienced were: exhaustion, back pain, painful nipples, cracked nipples, perineal pain, haemorrhoids, diarrhoea, constipation, and headache.	66.6 % (Medium)
Ayers et al.^46^ 2019	England	Qualitative, cross-sectional study Methods: writing assignment about stressful events	148 primiparous and multiparous women aged 21–42 years	Factors that cause stress in women during pregnancy, childbirth and/or the postpartum period Degree to which the women were upset or stressed by the event (scale of 1–10)	Between 6 and 12 weeks	18.2% reported health problems immediately after birth: hemorrhage and incontinence. 35.8% reported struggles adjusting to a new life with the baby: difficulties coping with a new baby and parenting, juggling responsibilities, exhaustion, sleep deprivation, and loneliness. Other experiences included: pressure to breastfeed, feelings of fear, disappointment and guilt when considering stopping breastfeeding. 18.2% reported stress from changing relationships: physical and emotional distance from partner, frustration towards partner, and problems with the sexual relationship.	77.7 % (High)
Buurman and Lagro-Janssen^25^ 2013	The Netherlands	Qualitative, cross-sectional study Methods: semi-structured face-to-face interviews	26 primiparous and multiparous women aged 20–40 years	Presence of problems with the pelvic floor Knowledge of women about problems of the pelvic floor Thresholds for seeking medical attention for problems with the pelvic floor	1 month to 1 year	100% reported pelvic floor pain, 19/26 had sexual dysfunction, 17/26 had urinary incontinence,16/26 had hemorrhoids, and 13/26 had micturition problems other than urinary incontinence. Furthermore: prolapse, problems with defecation, vaginal and anal flatulence, feelings of shame about pelvic floor problems, loss of control over one’s own body, and insecurity about the changing body.	88.8 % (High)
Dunn et al.^62^ 2019	United States	Prospective cohort study Methods: questionnaire	288 primiparous women aged >18 years	Prevalence and intensity of pain in the upper back, pain in the lower back and/or in the pelvic girdle during pregnancy and postpartum	Between 6 and 10 weeks	75% had pain in the upper back, lower back or pain in the pelvic girdle. For 13%, the pain hindered the women in their daily life. The majority of women had pain in multiple areas.	37.5 % (Low)
Forster and McLachlan^56^ 2010	Australia	Qualitative, longitudinal study Methods: structured telephone interviews	889 primiparous women, mean age 28 years	Experiences of women with breastfeeding	Just after birth and at 6 months	Women’s experiences were: shame when breastfeeding in public, feelings of failure or guilt when not breastfeeding, breastfeeding was experienced as exhaustive, painful and/or difficult, and high societal pressure to breastfeed.	77.7 % (High)
George^63^ 2005	United States	Qualitative, cross-sectional study Methods: semi-structured face-to-face interviews	10 primiparous women aged 18–35 years	Experiences of women with pregnancy, childbirth and the postpartum period	Until 4 weeks	Women’s experiences were: feeling unprepared for challenges in the postpartum period, concerns about adaptation to a new situation, overwhelming responsibility, concerns about taking on multiple tasks, uncertainty, women indicated they experienced pain and discomfort which interfered with their ability to care for themselves and their baby, fatigue, and lack of sleep.	60.0 % (Medium)
Gianni et al.^47^ 2020	Italy	Prospective cohort study Methods: questionnaire	273 primiparous women, mean age 34.5 years	Experiences with breastfeeding by first-time mothers	3 months	Reasons for women to describe their breastfeeding experience as negative were: fatigue, breast problems, perception of limited milk supply, latching difficulties, being unsure about breastfeeding, feeling not adequately supported. 80% experienced difficulties during breastfeeding: painful breasts and/or nipples, and cracked nipples. Other experiences were: emotional exhaustion, concern, uncertainty, fear, anger, sadness, and disappointment.	50.0 % (Medium)
Ishikawa et al.^65^ 2011	Japan	Prospective cohort study Methods: EPDS (cut-off 8/9) and Stein’s scale (cut-off 7/ 8)	423 primiparous and multiparous women, mean age 31 years	Depressive symptoms Mood shortly after childbirth	First 5 days and 1 month	15.7% and 11.0% of women met criteria for babyblues on day 1 and day 5, respectively. 10.4% scored above the cut-off on the EPDS at 1 month and met criteria for postpartum depressive symptoms.	37.5 % (Low)
Lee^48^ 2007	England	Mixed methods, cross-sectional study Methods: face-to-face interviews (phase 1) and telephone interviews (phase 2)	Phase 1: 33 primiparous and multiparous women aged 22–40 years Phase 2: 503 primiparous and multiparous women	Phase 1: reasons for bottle feeding Phase 2: Experiences of women with feeding their baby in the first 6 months postpartum	Phase 1: between 0–3 months Phase 2: between 0–6 months	Experiences of women in general: difficulty breastfeeding, wanting to return to normal, feeling of control when bottle feeding, wanting their own bodies back, and exhaustion. 33% felt guilty about using formula and 32% had a sense of failure about not breastfeeding. 20% were worried about the effects of formula on their baby’s health. Other experiences were: societal pressure to breastfeed, too little knowledge about bottle feeding and where to find information. Experiences with breastfeeding were: painful, exhaustive, leaking breasts, and sleeping problems.	50.0 % (Medium)
Li et al.^64^ 2008	United States	Prospective cohort study Methods: questionnaires	1323 primiparous and multiparous women aged ≥18 years	Reasons why women stop breastfeeding in the first year postpartum	At 2, 3, 4, 5, 6, 7, 8, 9, 10.5 and 12 months	Reasons for women to stop breastfeeding: wanting to return to their usual diet (6.5%), wanting their own bodies back to themselves (14.7%), pumping milk no longer seemed worth the effort (18.2%), too inconvenient (15.6%), wanting to leave the baby for several hours at a time (15.3%), too many household duties (9.0%), not wanting to breastfeed in public (11.6%), wanting or needing the baby to be fed by someone else (16.8%), not being able/willing to pump or breastfeed at work (14.6%), baby had trouble sucking or latching on (19.2%). Also, sore, bleeding or cracked nipples (15.4%); engorgement (8.8%); infected or abscessed breasts (4.6%); too much leaking of milk (5.9%); too painful (11.3%); too tiring (12.2%); and not having enough milk (45.5%).	62.5 % (Medium)
Lupton^57^ 2000	Australia	Qualitative, longitudinal study Methods: semi-structured interviews	25 primiparous women aged 23–35 years and their partners	Experiences and conceptions surrounding several aspects of parenthood	Between 2–10 days, 4–6 weeks, 12–14 weeks and 5–6 months	Experiences of postpartum women: difficulty finding balance between domestic tasks, childcare and selfcare; difficulty giving up autonomy and freedom; leaking breasts in socially inappropriate situations; cracked nipples; and breastfeeding is experienced as painful, uncomfortable, exhausting and time-consuming.	70 % (Medium)
Miller^49^ 2007	United Kingdom	Qualitative, longitudinal study Methods: semistructured face-toface interviews	17 primiparous women, mean age 30 years	Expectations, experiences surrounding birth, transition to motherhood, information seeking and work intentions	Between 6–8 weeks and 8–9 months	Experiences of postpartum women: loneliness, uncertainty, concern, feeling ill prepared for motherhood, mood swings, guilt about decisions surrounding childcare and return to work.	77.7 % (High)
Moossdorff-Steinhauser et al.50 2021	The Netherlands	Cross-sectional study Methods: questionnaires	415 primiparous and multiparous women, mean age 30.6 years	Beliefs, prevalence and severity of urinary incontinence, quality of life, and help-seeking behavior	Between 6 weeks and 3 months, 3–6 months or 6–12 months	57.1% of women experienced urinary incontinence in the first 12 months postpartum, of which for 38% the condition was bothersome. The highest prevalence was reported between 6 weeks and 3 months postpartum (66.7%). Women reported significant bother on physical activities, need to change underclothes, and worrying because of smell.	42.9% (Medium)
Mulherin and Johnstone^58^ 2015	Australia	Qualitative, cross-sectional study Methods: semi-structured interviews	12 primiparous women aged 15–24 years^a^	Experiences with transition to motherhood, experienced difficulties and joyous aspects	At one moment in the first 12 postpartum months	Experiences of postpartum women: loss of freedom and independence, exhaustion, lack of sleep, changes in relationship with partner, lack of social support, and negative self-image.	66.6 % (Medium)
Navodani et al.^59^ 2019	Australia	Prospective cohort study Methods: questionnaires or telephone interviews	1358 primiparous women, aged ≥18 years (1115 Australian-born and 243 migrant women from a non-English speaking background)^b^	Prevalence of common physical, emotional and social health problems during pregnancy until 18 months postpartum	At 3, 6, 9, 12 and 18 months	Experiences of postpartum women: extreme tiredness/exhaustion, back pain, hemorrhoids, constipation, breast problems, cough/cold, headache/migraine, pelvic pain, fecal incontinence, anxiety and depressive symptoms. 42.8% reported relationship problems during the first 12 months postpartum and 16.9% experienced some form of intimate partner abuse.	66.6 % (Medium)
O’Malley et al.^20^ 2018	Ireland	Prospective cohort study Methods: questionnaires	832 primiparous women aged ≥18 years	Prevalence of sexual health issues at 6 and 12 months postpartum and factors associated with postpartum sexual health issues	At 3, 6, 9 and 12 months	Experiences of postpartum women: At 6 months postpartum, 38.7% experienced dyspareunia, 42.8% lack of vaginal lubrication, and 50.9% loss of interest in sexual activity. At 12 months postpartum, 21.4% experienced dyspareunia, 32.5% lack of vaginal lubrication, and 39.2% loss of interest in sexual activity.	62.5 % (Medium)
O’Reilly et al.^60^ 2009	Australia	Qualitative, cross-sectional study Methods: face-to-face in-depth interviews	10 primiparous and multiparous women aged 25–35 years with persistent pelvic problems	Recovery experiences in the presence of continued pelvic problems in the puerperium	At one moment between 6 weeks and 4 years postpartum	Experiences of postpartum women: hemorrhoids, urinary incontinence, vaginal prolapse, bowel problems, dyspareunia, vaginal and perineal pain, bladder prolapse, and rectovaginal fistula. Experiences with pelvic problems: fearing intimacy, negative self-image, feelings of failure and guilt, and feelings of dismissal by healthcare providers.	80.0 % (High)
Olde et al.^51^ 2005	Netherlands	Prospective cohort study Methods: questionnaires	140 primiparous and multiparous aged 22–40 years	PTSD symptoms related to childbirth	During the first week and at 3 months	Experiences of postpartum women: 31.4% reported symptoms on one of the clusters of PTSD, 42.1% reported symptoms on two clusters of PTSD, and 10.7% on all three clusters. Most reported symptom: hyperarousal. Total prevalence of PTSD: 2.1%.	62.5 % (Medium)
Schmied and Lupton^61^ 2001	Australia	Qualitative, longitudinal study Methods: semistructured interviews	25 primiparous women aged 23–35 years	Expectations and experiences of first-time motherhood	Between 2–10 days, 4–6 weeks, and 5–6 months	Experiences of postpartum women: breastfeeding is exhausting, changes in appearance and sensation of breasts, alienation between self and body, wanting their ‘body back’, loss of self and agency, painful breasts, engorgement, cracked nipples, mastitis, limited milk supply, and leaking breast milk.	60.0 % (Medium)
Stomp-van den Berg et al.^52^ 2012	Netherlands	Prospective cohort study Methods: questionnaires	548 primiparous and multiparous women, mean age 32 years	Presence of pelvic girdle pain at 12 weeks postpartum, pain intensity and predictors	At 6 and 12 weeks	Prevalence of pelvic girdle pain: 48% between 0 and 6 weeks, and 43% between 6 and 12 weeks. Median pain intensity remained stable throughout pregnancy and postpartum.	62.5 % (Medium)
Thompson et al.^10^ 2002	Australia	Prospective cohort study Methods: questionnaire and EPDS (cut-off 12)	1193 primiparous and multiparous women aged ≥16 years	Presence of 12 parental health problems and depressive symptoms and resolution of these problems	At 8, 16 and 24 weeks	Experiences of postpartum women: exhaustion, backache, hemorrhoids, lack of sleep, sore perineum, excessive/prolonged bleeding, headaches/migraines, sexual problems, mastitis, bowel problems, urinary incontinence, and other urinary problems. 10 % of women experienced depressive symptoms.	62.5 % (Medium)
Van Brummen et al.^53^ 2006	Netherlands	Prospective cohort study Methods: Dutch translation of the Urogenital Distress Inventory questionnaire	344 primiparous women, mean age 30.4 years	Presence of urogenital symptoms during pregnancy and postpartum	At 3 and 12 months	11.4% experienced bothersome stress urinary incontinence at 12 months postpartum. 6.5% experienced bothersome urinary incontinence at 12 months postpartum.	75.0 % (High)
Wesselhoeft et al.^54^ 2020	Denmark, Tanzania and Vietnam	Cross-sectional study Methods: EPDS (cut-off 12)	4516 primiparous and multiparous women, mean age 28.4 years, (2069 women from Denmark (mean age 30.4 years), 1278 from Vietnam and 1169 from Tanzania)^c^	Presence of postpartum depressive symptoms	Denmark: at 3 months Vietnam and Tanzania: at 40 days	6.4% had a total EPDS score above cut-off 12 and therefore met the criteria for postpartum depressive symptoms. 35% experienced worry, 7.4% experienced fear, 9.0% experienced sadness, 0.9% thought about self-harm, and 5.1% experienced difficulty sleeping, 39.1% experienced self-blame, 3.9% experienced crying, and 21.9% felt overwhelmed.	66.6 % (Medium)

Quality assessment: 0–40% = low quality, 41–70% = medium quality, 71–100%= high quality. NA: not applicable.

a Of 12 study participants, two women were aged <18 years. Data from these are not women included in this table.

b Only data from women residing in Australia on health problems experienced up to 12 months postpartum are included.

c Only data from women residing in Denmark are included. EPDS: Edinburgh Postnatal Depression Scale.

Seven studies described women’s experiences regarding breastfeeding or bottle-feeding^45,47,48,55,56,61,64^. Seven studies examined physical health problems experienced, namely: problems with the pelvic floor^25,60^, back pain or pelvic girdle pain^52,62^, sexual problems^20^, and urinary incontinence^50,53^. Three studies described mental health problems experienced, namely postpartum depressive symptoms^54,65^ and posttraumatic stress disorder (PTSD)^51^. Five studies described women’s experiences during their transition to parenthood^46,49,57,58,63^. Finally, three studies focused on the prevalence of multiple health problems and challenges postpartum^4,10,59^.

### Results of the quality assessment

Results of the quality assessment are shown in Tables 2–4. The majority of the studies were rated medium (n=15) or high quality (n=8). Two cohort studies were rated low quality^62,65^ due to: unclear reporting of the population or outcome measures, failure to identify or investigate confounding factors, and insufficient explanation of the strategies used for dealing with incomplete follow-up. Both low-quality articles were excluded from further data synthesis.

**Table 2:** Quality assessment for the included qualitative studies and mixed-methods studies, scored according to a study design specific checklist^41^

*Authors Year*	*Congruity between philosophical perspective and methodology*	*Congruity between methodology and research question*	*Congruity between methodology and methods for data collection*	*Congruity between methodology and analysis of data*	*Congruity between methodology and interpretation of results*	*Cultural or theoretical statement by the researcher*	*Influence of researcher on research, and vice-versa, addressed*	*Participants and their voice adequately represented*	*Ethical research or ethical approval*	*Conclusions drawn from analysis or interpretation of the data*	*Total score (quality assessment)*
Alianmoghaddam et al.^55^ 2017	Yes	Yes	Yes	Yes	Yes	No	No	Yes	Yes	Yes	80% (High)
Andrew and Harvey^45^ 2011	NA	Yes	Yes	Yes	Yes	No	No	Yes	Yes	Yes	77.7% (High)
Ayers et al.^46^ 2019	NA	Yes	Yes	Yes	Yes	No	No	Yes	Yes	Yes	77.7% (High)
Buurman and Lagro-Janssen^25^ 2013	NA	Yes	Yes	Yes	Yes	No	Yes	Yes	Yes	Yes	88.8% (High)
Forster and McLachlan^56^ 2010	NA	Yes	Yes	Yes	Yes	No	No	Yes	Yes	Yes	77.7% (High)
George^63^ 2005	Yes	Yes	Yes	Yes	Yes	No	No	Unclear	Yes	Unclear	60.0% (Medium)
Lee^48^ 2007	Yes	Unclear	Unclear	Yes	Yes	No	No	Yes	Unclear	Yes	50.0 % (Medium)
Lupton^57^ 2000	Yes	Yes	Yes	Yes	Yes	No	No	Yes	No	Yes	70% (Medium)
Miller^49^ 2007	NA	Yes	Yes	Yes	Yes	No	Yes	Yes	Unclear	Yes	77.7% (High)
Mulherin and Johnstone^58^ 2015	NA	Yes	Yes	Yes	Yes	No	Unclear	Yes	Unclear	Yes	66.6% (Medium)
O’Reilly et al.^60^ 2009	Yes	Yes	Yes	Yes	Yes	No	Unclear	Yes	Yes	Yes	80.0% (High)
Schmied and Lupton^61^ 2001	Yes	Yes	Yes	Unclear	Yes	No	No	Yes	No	Yes	60.0% (Medium)

Quality assessment: 0–40% = low quality, 41–70% = medium quality, 71–100%= high quality. NA: not applicable.

**Table 3:** Quality assessment for the included prospective cohort studies, scored according to a study design specific checklist^42^

*Authors Year*	*Two groups similar and recruited from same population*	*Exposures measured similarly to assign women to groups*	*Exposure measured in valid and reliable way*	*Identification of confounding factors*	*Statement of strategies to deal with confounding factors*	*Groups or participants free of outcome at start of study*	*Outcomes measured in valid and reliable way*	*Followup time reported and sufficiently long*	*Follow-up complete or reasons to loss to follow-up explored*	*Use of strategies to address incomplete follow-up*	*Appropriate statistical analysis*	*Total score (quality assessment)*
Dunn et al.^62^ 2019	NA	NA	NA	No	No	Unclear	Unclear	Yes	Yes	No	Yes	37.5 % (Low)
Gianni et al.^47^ 2020	NA	NA	NA	No	Unclear	Yes	Unclear	Yes	No	Yes	Yes	50.0 % (Medium)
Ishikawa et al.^65^ 2011	NA	NA	NA	Yes	Unclear	No	Yes	Unclear	No	No	Yes	37.5 % (Low)
Li et al.^64^ 2008	NA	NA	NA	Yes	Yes	Yes	Unclear	Yes	No	No	Yes	62.5 % (Medium)
Navodani et al.^59^ 2019	Unclear	NA	NA	Yes	Yes	Unclear	Yes	Yes	Yes	No	Yes	66.6 % (Medium)
O’Malley et al.^20^ 2018	NA	NA	NA	Yes	Yes	No	Yes	Yes	No	No	Yes	62.5 % (Medium)
Olde et al.^51^ 2005	NA	NA	NA	Yes	Yes	Unclear	Yes	Yes	Unclear	No	Yes	62.5 % (Medium)
Stomp-van den Berg et al.^52^ 2012	NA	NA	NA	Yes	Yes	No	No	Yes	Yes	No	Yes	62.5 % (Medium)
Thompson et al.^10^ 2002	NA	NA	NA	Yes	Yes	No	Unclear	Yes	Yes	Unclear	Yes	62.5 % (Medium)
Van Brummen et al.^53^ 2006	NA	NA	NA	Yes	Yes	Unclear	Yes	Yes	No	Yes	Yes	75 % (High)

Quality assessments: 0–40% = low quality, 41–70% = medium quality, 71–100% = high quality. NA: not applicable.

**Table 4:** Quality assessment for the included cross sectional studies and retrospective studies, scored according to a study design specific checklist^42,43^

*Authors Year*		*Clearly defined criteria for inclusion*	*Detailed description of study subjects*	*Exposure measured in valid and reliable way*	*Use of objective, standard criteria for measurement of the condition*	*Identification of confounding factors*	*Statement of strategies to deal with confounding factors*	*Outcomes measured in a valid and reliable way*	*Appropriate statistical analysis*	*Total score (quality assessment)*
Moossdorff-Steinhauser^50^ 2021		Unclear	No	NA	Yes	No	No	Yes	Yes	42.9% (Medium)
Wesselhoeft et al.^54^ 2020		Unclear	No	NA	NA	Yes	Yes	Yes	Yes	66.6 % (Medium)
***Authors Year***	***Appropriate sample frame***	***Appropriate sampling of study participants***	***Adequate sample size***	***Detailed description of study subjects and setting***	***Data analysis conducted with sufficient coverage of identified sample***	***Use of valid methods for identification of the condition***	***Condition measured in a standard, reliable way for all participants***	***Appropriate statistical analysis***	***Adequate response rate or appropriate management of low response rate***	***Total score (quality assessment)***
Ansara et al.^4^ 2005	Yes	Yes	Unclear	Yes	No	No	Yes	Yes	Yes	66.6 % (Medium)

Quality assessments: 0–40% = low quality, 41–70% = medium quality, 71–100% = high quality. NA: not applicable.

### Results of data-extraction and synthesis

An overview of all health problems that women experienced during the first year postpartum (n=83) is shown in Table 5. A number of postpartum health problems emerged that are not described in the ICD-10^44^ and therefore could not be classified into any of the aforementioned categories. Consequently, two categories were added to the overview: ‘problems related to feeding the infant’ and ‘other challenges’.

**Table 5:** Overview of the identified health problems and challenges during the first year postpartum

*Main category*	*Subcategory*	*Identified health problems experienced by women*
**Physical health problems**	General health problems	Back pain Constipation Cough/common cold Diarrhea Excessive bleeding (Extreme) exhaustion Headache Pelvic girdle pain
	Problems related to the pelvic floor and urogenital system	Anal flatulence Bladder prolapse Dyspareunia Fecal incontinence Hemorrhoids Perineal pain Rectovaginal fistula Urinary incontinence Other urinary problems (frequency, voiding difficulties) Vaginal flatulence Vaginal prolapse
	Breast problems	Altered appearance and sensation of breasts Breast abscess Engorgement Leakage of breastmilk Mastitis Painful breasts Painful/cracked nipples
**Mental health problems**	Psychiatric disorders	Anxiety Depressive symptoms Difficulty sleeping Mood swings Posttraumatic Stress Disorder (PTSD)
	Sexual issues	Fear of intimacy Lack of vaginal lubrication Loss of sexual interest
**Social health problems**	Problems in relationships	Changed relationship with family members Intimate partner abuse Lack of social support Relationship issues
**Problems related to feeding the infant**	Problems associated with breastfeeding	Breastfeeding is exhausting and time-consuming Difficulty expressing milk Difficulty latching the baby onto the nipple Ineffective milk transfer Limited milk supply Societal pressure to breastfeed
	Problems surrounding infant feeding choices	Concerns about long-term consequences of formula feeding Feelings of failure when unable to breastfeed Feelings of guilt when unable to breastfeed Insufficient information on formula feeding Judgement of others about feeding choices
	Breastfeeding in public	Experienced inconvenience or embarrassment of expressing milk at work Feeling embarrassed breastfeeding in front of others Feeling isolated at home because of breastfeeding Feeling uncomfortable breastfeeding in public Lack of public spaces for breastfeeding
	Insufficient knowledge and/or preparation	Feeling unprepared for breastfeeding challenges/issues
**Other challenges**	Feelings and emotions	Anger Concern/worry Crying Difficulty adapting to a new life as a mother Difficulty coping with the baby crying Disappointment Emotional exhaustion Fear Feeling overwhelmed Feeling unprepared for the postpartum period Feelings of failure/self-blame for loss of satisfying sexual relationships Feelings of guilt for returning to work Feelings of shame because of pelvic floor problems Loneliness Loss of control over own body Loss of independence Negative self-image Neglect of pelvic floor problems because of shame Sadness Self-blame Uncertainty Wanting to regain control of their body
	Practical challenges	Difficulty dividing attention between children Difficulty juggling responsibilities Feelings of dismissal by healthcare providers Insufficient time for self-care

### Identified health problems

*Physical health problems*

The most commonly described physical health problem was (extreme) exhaustion. The prevalence of exhaustion as experienced by women between 8 and 12 weeks postpartum varied from 46% to 66.3%^4,10,59^. At six months postpartum, prevalences between 48% and 59.9% have been reported^10,59^. Back pain was described by three studies^4,10,59^. The prevalence of back pain varied between 53% and 60.5% at 8 and 12 weeks postpartum, respectively^4,10,59^, and between 43% and 58.9% at six months postpartum^10,59^. Other physical health problems experienced were: headache^4,10,59^, pelvic girdle pain^52-59^, constipation^4,10,59^, diarrhea^4,10^, excessive bleeding^10^, cough and/or the common cold^59^.

Urinary incontinence was the most frequently mentioned health problem related to the pelvic floor and urogenital system^10,25,50,53^. The reported prevalence varied from 21.0% in the first two months postpartum to 10.5% at 12 months postpartum^10,53^. Some studies reported a total prevalence of 57.1% and 64.4% during the first year postpartum^25,50^. A distinction was made between stress urinary incontinence and urge urinary incontinence in several studies^50,53^.

Hemorrhoids were frequently mentioned and reported prevalences varied from 35.5% in the first 8 weeks postpartum to 11.8% at 12 months postpartum^45,59^. Other pelvic floor problems were: fecal incontinence, perineal pain, other urinary problems such as frequency or voiding difficulties, and sexual health problems such as dyspareunia. The prevalence of dyspareunia was 38.7% and 21.4% at 6 and 12 months postpartum^20^.

Problems related to the breasts included painful or cracked nipples, and painful breasts^4,45,47,48,55-57,61,64^. Additionally, leakage of milk from the breasts at socially inappropriate times was mentioned^48,57,61,64^. For some women, these complaints were reasons to consider the cessation of breastfeeding^45,48,55,64^. Experienced complications related to lactation, such as mastitis and breast abscess, show a decreasing trend in prevalence from 15% in the first 2 months postpartum to 3.1% at 9 months postpartum^10,61,64^.

*Mental health problems*

The reported prevalence of postpartum depressive symptoms varied between 6.4% and 7.0% at three months postpartum^54,59^, and between 8% and 8.9% at six months postpartum^10,59^. In addition, Navodani et al.^59^ found the highest percentage (15.4%) of women experiencing anxiety or panic attacks at 3 months postpartum. One study reported that 2.1% of studied participants met the criteria for PTSD up to 3 months postpartum, according to the Diagnostic and Statistical Manual of Mental Disorders, fourth edition (DSM-IV)^51^. Other health problems mentioned in Chapter V of the ICD-10 that were found in this study included sexual problems such as insufficient vaginal lubrication (42.8%) and loss of interest in sexual intercourse (50.9%) at 6 months postpartum^20^. Another commonly experienced problem was difficulty sleeping^10,46,48,54,63^. Thompson et al.^10^ reported a prevalence of 28% at 2 months and 2% at 6 months postpartum.

*Social health problems*

Social health problems included stress, lack of social support, and altered relationships with partners, family members and other children^46,47,57-59,63^. Navodani et al.^59^ examined intimate partner abuse and found that 16.9% of women experienced some form of abuse in the first 12 months postpartum.

*Problems related to feeding the infant*

Breastfeeding has been described as exhausting and time consuming^45,47,48,56,57,61,64^. Common breastfeeding problems included experiencing difficulty latching the baby onto the nipple and difficulty expressing milk^45,47,64^. Several women felt that their milk supply was insufficient^45,47,55,61,64^. For some, this was a reason for switching to formula feeding^55^.

Societal pressure to breastfeed has been described as an influential factor in deciding between breastfeeding and formula feeding^45,46,48,55,56^. Among women who failed their breastfeeding goals, some felt guilty or ashamed when switching to formula feeding^45,46,48,55,56^.

Several women felt ashamed, embarrassed, fearful or uncomfortable while breastfeeding in public^45,56,64^. Li et al.^64^ showed that for 11.6% of women this was a reason to quit breastfeeding. A lack of public spaces for breastfeeding restricted some women from leaving their homes^56^. In addition, some women indicated that they were not willing or unable to express breastmilk at work out of embarrassment^45,64^.

*Other challenges*

Some women felt they lost their independence and freedom since the birth of their baby^45,57,58^. Some felt insufficiently prepared for the realities and challenges of the postpartum period^49,55,63^. The severity of health problems and the overwhelming responsibility of motherhood was often underestimated^49,63^. Loneliness^46,49^, fear^47,63^, uncertainty^25,49,63^, concern^47,49,54^, guilt^49,60^, and a negative self-image^58,60^, are examples of emotions and feelings experienced by women in the first year postpartum. Some women found it difficult to balance various responsibilities, such as housekeeping, caring for other children and caring for themselves^25,45,46,57,64^.

## DISCUSSION

In this review, a significant number of health problems that women experience during the first year postpartum (n=83) were identified. These health problems varied from physical, mental and social problems, to problems with breastfeeding and practical challenges of motherhood. Examples of health problems commonly experienced are: exhaustion, back pain, pelvic floor problems, sexual problems, relationship problems, and feelings of failure.

Health problems experienced by women from high-income countries were identified. The continents Europe, Oceania, and North America were best represented. No high- or medium-quality studies were found that focused on women from high-income countries in Asia, the Middle East or South America, indicating a gap in the literature regarding health problems experienced by these women. Additionally, in the majority of the included studies (n=15), most women finished secondary school or were educated to a degree level^4,20,25,46,47,50,52-57,59,61,63^. A possible explanation for this is that women with lower levels of education are less likely to participate in scientific research^66-68^.

Several studies present prevalences of reported health problems, some of which were mentioned in the results section of this article. When briefly analyzing these prevalences, it appears that reported prevalences of most health problems do not substantially differ between the included studies. More substantial differences in prevalence between studies might be explained by the lack of clear definitions of health problems, different time point measurements within the first year postpartum or the use of different questionnaires in the identification of a health problem. Additionally, even though this study reports health problems experienced by women from high-income countries, it is important to acknowledge that, between these countries, disparities exist in healthcare services that could influence the reported prevalences of postpartum health problems.

Difficulty sleeping and several sexual health problems were categorized as mental health problems in this article, in order to ensure continuity in the classification according to the ICD-10^44^. Although these health problems can be symptoms of a mental health disorder, the authors acknowledge that they can also be a consequence of factors such as nightly infant feeding or wakefulness, or pelvic floor problems.

Even though several prevalences of identified postpartum health problems are presented in this review, it should be noted that the included studies used different methods for investigating health problems experienced, measured the presence of health problems at various time points, and sometimes failed to mention the influence of confounding factors such as age and socioeconomic background.

### Implications for practice and/or policy

The overview of health problems experienced by women in the first year postpartum presented in this review can be meaningful in several ways, both in practice and in healthcare education.

*In the healthcare setting*

Poor parental health negatively influences women’s health, functioning, parenting behavior, and children’s health outcomes^4-12^. Therefore, it seems imperative that healthcare providers pay attention to the possible health issues women might experience postpartum and explore their individual needs regarding medical treatment, in accordance with recommendations by the World Health Organization and The American College of Obstetricians and Gynecologists^69,70^. The overview of health problems and challenges presented in this review could serve as a useful tool for healthcare professionals. It can be used during antenatal and postpartum consultations to discuss postpartum challenges, such as pelvic floor problems, emotional challenges such as fear and anxiety, and problems related to breastfeeding.

*Education*

Previous studies have shown that women have insufficient knowledge of possible postpartum health problems and that they feel insufficiently prepared for potential challenges^33,34,71^. Several articles, found in this systematic review, confirm this lack of knowledge and preparation^25,49,55,63^. To be able to better educate and prepare women, the information on common health problems and challenges identified in this review can be used to develop educational material or interventions. For example, a brochure on common postpartum health problems could serve as prepartum preparation for potential future challenges^71^. Furthermore, the information obtained in this systematic review can serve as a basis for the development of openly accessible information sources, such as online courses, that can help women who have given birth to look up reliable information and be informed during the postpartum period^72^.

*In education of healthcare*

The overview of postpartum health problems experienced can be a meaningful contribution to the education of healthcare professionals. Receiving education on health problems that women can experience postpartum can lead to application of this information later on in their careers.

### Further research

A gap in knowledge has been identified in the literature on women’s experiences with mental health problems in the first year postpartum. Further research into the experiences and needs of these women using specifically qualitative methods is recommended, so that postpartum care can be optimized. Lastly, it is possible that women from high-income countries experience other health problems and challenges in the first year postpartum than women from low-income countries. Additional research might identify key distinctions between these groups that could be used to improve postpartum care in low-income countries.

### Strengths and limitations

The current review distinguishes itself from previous reviews, because it focuses on women’s experiences regarding postpartum health problems. Previous reviews have mostly summarized evidence on these problems from an objective point of view and have not researched subjectively experienced problems^28-31^. Furthermore, unique to this review is the inclusion of problems regarding infant feeding, social, emotional, and practical problems and challenges, besides mental and physical problems. In comparison to previous studies^4,10,13,32-35^, the overview presented in this review is therefore a more diverse and complete presentation of health problems that women can experience in the first year postpartum.

One of the strengths of this review is that most items on the PRISMA checklist for systematic reviews were carried out independently by two assessors^36^. Additionally, to identify as many relevant articles as possible, both reference lists and citations of all included articles were screened for eligibility. This screening resulted in several relevant (n=13) studies and therefore the chances of missing important articles were minimized. Only medium to high quality articles were used for data-synthesis. Low quality articles were excluded, as they may have adversely affected the reliability of the results.

Another strength of this review is that the search strategy was focused on postpartum experiences from women residing in high-income countries all over the world. The health problems identified in this review are thus representative for women from several different continents.

Several potential limitations of this study should be considered. Most women that participated in the included studies were well educated, therefore the current study might not be representative for all women across the full range of socioeconomic backgrounds. Furthermore, the influence of parity on health problems experienced during the first year postpartum was not investigated, even though parity is a potential confounding factor. Due to the exclusion of studies exclusively reporting data from women with preexisting medical conditions, women who used reproductive techniques for conception and women who experienced a high-risk situation during pregnancy or childbirth, several postpartum health problems experienced by these women may be missing from the overview presented in this review.

The reviewers did not seek contact with authors and did not search for unpublished articles and grey literature during the screening process. Clarifying information or relevant data on health problems experienced may have been missed because of this. However, a large number of health problems were identified in this systematic review, implying that the most prevalent health problems experienced by women during the first year postpartum were found.

## CONCLUSIONS

The information obtained from this systematic review and the updated overview of postpartum health problems contribute to a wider understanding of women’s postpartum health. More awareness among women and healthcare professionals about the health problems women may experience after pregnancy and childbirth, can ensure that these issues are discussed more openly during postpartum consultations. This could lead to improvement of postpartum healthcare for women and corresponds to the principles of PCC in which care is adjusted to the women’s needs^22-24^.

## Data Availability

Data sharing is not applicable to this article as no new data were created.
