# Corrigendum: Health problems experienced by women during the first year postpartum: A systematic review

**DOI:** 10.18332/ejm/181537

**Published:** 2024-02-05

**Authors:** Marije M. Gmelig Meyling, M. Evelyn Frieling, Johanna P. M. Vervoort, Esther I. Feijen-de Jong, Danielle E. M. C. Jansen

**Affiliations:** 1 University of Groningen, Groningen, the Netherlands; 2 Department of Health Sciences, University of Groningen, University Medical Center Groningen, Groningen, the Netherlands; 3 Department of Primary and Long-term Care, University of Groningen, University Medical Center Groningen, Groningen, the Netherlands; 4 Department of Midwifery Science, Amsterdam University Medical Centers, Vrije Universiteit Amsterdam, Amsterdam Public Health Research Institute, Amsterdam, Amsterdam, the Netherlands; 5 Midwifery Academy Amsterdam Groningen, Inholland, Groningen, the Netherlands

**Keywords:** maternal health, postpartum period, exhaustion, urinary incontinence, depressive symptoms, systematic review

Corrigendum on:

Health problems experienced by women during the first year postpartum: A systematic review

By Marije M. Gmelig Meyling, M. Evelyn Frieling, Johanna P. M. Vervoort, Esther I. Feijen-de Jong, Danielle E. M. C. Jansen

European Journal of Midwifery, Volume 7, Issue December, Pages 1-20, Publish date: 18 December 2023

DOI: https://doi.org/10.18332/ejm/173417

In the originally published version of the article, the first author's name was incorrect on the footer area-copyright and is now corrected to Gmelig Meyling M.M. et al.

Furthermore, the same author's initials in the Authors’ Contributions section are also corrected to MMGM. These are also corrected online.

