# Supplementary material for: Health problems experienced by women during the first year postpartum: A systematic review
Source: Eur J Midwifery. 2023 Dec 18;7:42. doi: 10.18332/ejm/173417 (PMC10726257; doi:10.18332/ejm/173417)
Supplement: Supplementary file 1 [file EJM-7-42-s1.pdf]

SUPPLEMENTARY FILE

A: Reporting Guideline checklist: PRISMA 2009 Checklist for 'Health problems experienced by people during the first year postpartum: a systematic review' (2023).

Section/topic	#	Checklist item	Reported on page #
**TITLE**			
Title	1	Identify the report as a systematic review, meta-analysis, or both.	Title, 1-4
**ABSTRACT**			
Structured summary	2	Provide a structured summary including, as applicable: background; objectives; data sources; study eligibility criteria, participants, and interventions; study appraisal and synthesis methods; results; limitations; conclusions and implications of key findings; systematic review registration number.	1
**INTRODUCTION**			
Rationale	3	Describe the rationale for the review in the context of what is already known.	1,3-4
Objectives	4	Provide an explicit statement of questions being addressed with reference to participants, interventions, comparisons, outcomes, and study design (PICOS).	3-5
**METHODS**			
Protocol and registration	5	Indicate if a review protocol exists, if and where it can be accessed (e.g., Web address), and, if available, provide registration information including registration number.	4
Eligibility criteria	6	Specify study characteristics (e.g., PICOS, length of follow-up) and report characteristics (e.g., years considered, language, publication status) used as criteria for eligibility, giving rationale.	3-5
Information	7	Describe all information sources (e.g., databases with dates of coverage, contact with study authors to	4-5

sources		identify additional studies) in the search and date last searched.	
Search	8	Present full electronic search strategy for at least one database, including any limits used, such that it could be repeated.	Appendix B
Study selection	9	State the process for selecting studies (i.e., screening, eligibility, included in systematic review, and, if applicable, included in the meta-analysis).	4-5
Data collection process	10	Describe method of data extraction from reports (e.g., piloted forms, independently, in duplicate) and any processes for obtaining and confirming data from investigators.	5-6
Data items	11	List and define all variables for which data were sought (e.g., PICOS, funding sources) and any assumptions and simplifications made.	4-5
Risk of bias in individual studies	12	Describe methods used for assessing risk of bias of individual studies (including specification of whether this was done at the study or outcome level), and how this information is to be used in any data synthesis.	6
Summary measures	13	State the principal summary measures (e.g., risk ratio, difference in means).	Not applicable
Synthesis of results	14	Describe the methods of handling data and combining results of studies, if done, including measures of consistency (e.g., I^2) for each meta-analysis.	5-6

**Section/topic**	**#**	**Checklist item**	**Reported on page #**
Risk of bias across studies	15	Specify any assessment of risk of bias that may affect the cumulative evidence (e.g., publication bias, selective reporting within studies).	6

Additional analyses	16	Describe methods of additional analyses (e.g., sensitivity or subgroup analyses, meta-regression), if done, indicating which were pre-specified.	Not applicable
**RESULTS**			
Study selection	17	Give numbers of studies screened, assessed for eligibility, and included in the review, with reasons for exclusions at each stage, ideally with a flow diagram.	6-7, Figure A
Study characteristics	18	For each study, present characteristics for which data were extracted (e.g., study size, PICOS, follow-up period) and provide the citations.	6-7, Table A
Risk of bias within studies	19	Present data on risk of bias of each study and, if available, any outcome level assessment (see item 12).	7, Tables B-D
Results of individual studies	20	For all outcomes considered (benefits or harms), present, for each study: (a) simple summary data for each intervention group (b) effect estimates and confidence intervals, ideally with a forest plot.	8-10
Synthesis of results	21	Present results of each meta-analysis done, including confidence intervals and measures of consistency.	Not applicable
Risk of bias across studies	22	Present results of any assessment of risk of bias across studies (see Item 15).	7, Tables B-D
Additional analysis	23	Give results of additional analyses, if done (e.g., sensitivity or subgroup analyses, meta-regression [see Item 16]).	Not applicable
**DISCUSSION**			
Summary of evidence	24	Summarize the main findings including the strength of evidence for each main outcome; consider their relevance to key groups (e.g., healthcare providers, users, and policy makers).	10-12

Limitations	25	Discuss limitations at study and outcome level (e.g., risk of bias), and at review-level (e.g., incomplete retrieval of identified research, reporting bias).	12-13
Conclusions	26	Provide a general interpretation of the results in the context of other evidence, and implications for future research.	10-12,14
**FUNDING**			
Funding	27	Describe sources of funding for the systematic review and other support (e.g., supply of data); role of funders for the systematic review.	15, Title page

From: Moher D, Liberati A, Tetzlaff J, Altman DG, The PRISMA Group (2009). Preferred Reporting Items for Systematic Reviews and Meta-Analyses: The PRISMA Statement. PLoS Med 6(7): e1000097. doi:10.1371/journal.pmed1000097

For more information, visit: **www.prisma-statement.org**

B: Search strategy, adapted to fit each electronic database for 'Health problems experienced by people during the first year postpartum: a systematic review' (2023).

Description: Complete search strategies performed in electronic databases MEDLINE, CINAHL and PsycINFO, after being tested and adapted to fit each electronic database.

<u>MEDLINE</u>

("Puerperal Disorders"[Mesh] OR "Maternal Health"[Mesh] OR "Health"[Mesh] OR "Health status"[Mesh] OR "Pathological Conditions, Signs and Symptoms"[Mesh] OR "Female Urogenital Diseases and Pregnancy Complications"[Mesh] OR puerperal disorder*[tiab] OR health[tiab] OR female urogenital disease*[tiab] OR symptom*[tiab] OR disease*[tiab] OR well-being[tiab] OR wellbeing[tiab] OR stress*[tiab] OR need*[tiab] OR transition*[tiab] OR concern*[tiab] OR challeng*[tiab] OR issue*[tiab] OR difficult*[tiab] OR psychologic*[tiab] OR social*[tiab])

AND

("Perception"[Mesh] OR perceive*[tiab] OR perception*[tiab] OR perspective[tiab] OR experienc*[tiab] OR insight[tiab] OR view*[tiab] OR imag*[tiab] OR dissatisfied[tiab] OR dissatisfy*[tiab] OR dissatisfaction[tiab])

AND

("Netherlands"[Mesh] OR netherland* OR dutch* OR utrecht OR erasmus OR amsterdam OR groningen OR leiden OR rotterdam OR maastricht OR radboud OR nijmegen OR dutch*[tiab] OR western countr*[tiab] OR western soci*[tiab] OR western cult*[tiab] OR high income countr*[tiab] OR high income popu*[tiab] OR western world[tiab] OR high socioeconomic background*[tiab])

AND

("Mothers"[Mesh] OR "Women"[Mesh] OR "Pregnant Women"[Mesh] OR pregnant woma*[tiab] OR pregnant wome*[tiab] OR mother*[tiab] OR parent*[tiab] OR mom[tiab] OR motherhood[tiab] OR women*[tiab] OR maternity[tiab] OR maternal*[tiab])

AND

("Postpartum Period"[Mesh] OR postpartum[tiab] OR post-partum[tiab] OR puerperium[tiab] OR after birth[tiab] OR after childbirth*[tiab] OR after pregnan*[tiab] OR perinatal[tiab] OR motherhood[tiab] OR fourth trimester[tiab] OR child's first year[tiab])

May 26th 2020: 669 results
July 2nd (re-run): 757 results

<u>PsycINFO</u>

(DE "Postpartum Depression" OR DE "Health" OR DE "Health status+" OR DE "Symptoms" OR TI (puerperal disorder*) OR AB (puerperal disorder*) OR TI (health) OR AB (health) OR TI (female urogenital disease*) OR AB (female urogenital disease*) OR TI (symptom*) OR AB (symptom*) OR TI (disease*) OR AB (disease*) OR TI (well-being) OR AB (well-being) OR TI (wellbeing) OR AB (wellbeing) OR TI (stress*) OR AB (stress*) OR TI (need*) OR AB (need*) OR TI (transition*) OR AB (transition*) OR TI (concern*) OR AB (concern*) OR TI (challeng*) OR AB (challeng*) OR TI (issue*) OR AB (issue*) OR OR TI (difficult*) OR AB (difficult*) OR TI (psychologic*) OR AB (psychologic*) OR TI (social*) OR AB (social*))

AND

(DE "Perception" OR TI (perceive*) OR AB (perceive*) OR TI (perception*) OR AB (perception*) OR TI (perspective) OR AB (perspective) OR TI (experienc*) OR AB (experienc*) OR TI (insight) OR AB (insight) OR TI (view*) OR AB (view*) OR TI (imag*) OR AB (imag*) OR TI (dissatisfied) OR AB (dissatisfied) OR TI (dissatisfy*) OR AB (dissatisfy*) OR TI (dissatisfaction) OR AB (dissatisfaction))

AND

((netherland*) OR (dutch*) OR (utrecht) OR (erasmus) OR (amsterdam) OR (groningen) OR (leiden) OR (rotterdam) OR (maastricht) OR (radboud) OR (nijmegen) OR TI (dutch*) OR AB (dutch*) OR TI (western countr*) OR AB (western countr*) OR TI (western soci*) OR AB (western soci*) OR TI (western cult*) OR AB (western cult*) OR TI (high income countr*) OR AB (high income countr*) OR TI (high income popu*) OR AB (high income

popu*) OR TI (western world) OR AB (western world) OR TI (high socioeconomic background*) OR AB (high socioeconomic background*))

AND

(DE “Mothers” OR DE “Human Females” OR TI (pregnant woma*) OR AB (pregnant woma*) OR TI (pregnant wome*) OR AB (pregnant wome*) OR TI (mother*) OR AB (mother*) OR TI (parent*) OR AB (parent*) OR TI (mom) OR AB (mom) OR TI (motherhood) OR AB (motherhood) OR TI (women*) OR AB (women*) OR TI (maternity) OR AB (maternity) OR TI (maternal*) OR AB (maternal*))

AND

(DE “Postnatal Period” OR TI (postpartum*) OR AB (postpartum*) OR TI (post-partum*) OR AB (post-partum*) OR TI (puerperium) OR AB (puerperium) OR TI (after birth) OR AB (after birth) OR TI (after childbirth*) OR AB (after childbirth*) OR TI (after pregnan*) OR AB (after pregnan*) OR TI (perinatal) OR AB (perinatal) OR TI (motherhood) OR AB (motherhood) OR TI (fourth trimester) OR AB (fourth trimester) OR TI (child’s first year) OR AB (child’s first year))

May 26th 2020: 264 results
July 2nd 2021 (re-run): 297 results

<u>CINAHL</u>

(MH "Puerperal Disorders+" OR MH "Women’s Health+" OR MH "Health+" OR MH "Health Status+" OR MH "Signs and Symptoms+" OR MH "Female Urogenital Diseases and Pregnancy Complications+" OR TI “puerperal disorder*” OR AB “puerperal disorder*” OR TI health OR AB health OR TI “female urogenital disease*” OR AB “female urogenital disease*” OR TI “symptom*” OR AB “symptom*” OR TI “disease*” OR AB “disease*” OR TI well-being OR AB well-being OR TI wellbeing OR AB wellbeing OR TI “stress*” OR AB “stress*” OR TI “need*” OR AB “need*” OR TI “transition*” OR AB “transition*” OR TI “concern*” OR AB “concern*” OR TI “challeng*” OR AB “challeng*” OR TI “issue*” OR AB “issue*” OR TI “difficult*” OR AB “difficult*” OR TI “psychologic*” OR AB “psychologic*” OR TI “social*” OR AB “social*”)

AND

(MH "Perception+" OR TI “perceive*” OR AB “perceive*” OR TI “perception*” OR AB “perception*” OR TI perspective OR AB perspective OR TI “experienc*” OR AB “experienc*” OR TI insight OR AB insight OR TI “view*” OR AB “view*” OR TI “imag*” OR AB “imag*” OR TI dissatisfied OR AB dissatisfied OR TI “dissatisfy*” OR AB “dissatisfy*” OR TI dissatisfaction OR AB dissatisfaction)

AND

(MH "Netherlands+" OR “netherland*” OR “dutch*” OR utrecht OR erasmus OR amsterdam OR groningen OR leiden OR rotterdam OR maastricht OR radboud OR nijmegen OR TI “dutch*” OR AB “dutch*” OR TI “western countr*” OR AB “western countr*” OR TI “western soci*” OR AB “western soci*” OR TI “western cult*” OR AB “western cult*” OR TI “high income countr*” OR AB “high income countr*” OR TI “high income popu*” OR AB “high income popu*” OR TI western world OR AB western world OR TI “high socioeconomic background*” OR AB “high socioeconomic background*”)

AND

(MH "Mothers+" OR MH "Women+" OR MH "Expectant Mothers" OR TI “pregnant woma*” OR AB “pregnant woma*” OR TI “pregnant wome*” OR AB “pregnant wome*” OR TI “mother*” OR AB “mother*” OR TI “parent*” OR AB “parent*” OR TI mom OR AB mom OR TI motherhood OR AB motherhood OR TI “women*” OR AB “women*” OR TI maternity OR AB maternity OR TI “maternal*” OR AB “maternal*”)

AND

(MH "Postnatal Period+" OR TI postpartum OR AB postpartum OR TI post-partum OR AB post-partum OR TI puerperium OR AB puerperium OR TI after birth OR AB after birth OR TI “after childbirth*” OR AB “after childbirth*” OR TI “after pregnan*” OR AB “after pregnan*” OR TI perinatal OR AB perinatal OR TI motherhood OR AB motherhood OR TI fourth trimester OR AB fourth trimester OR TI child’s first year OR AB child’s first year)

May 26th 2020: 246 results
July 2nd 2021 (re-run): 264 results

C: Data extraction form for 'Health problems experienced by people during the first year postpartum: a systematic review' (2023).

<u>Reference #</u>

Title and author:
Year of publication:
Journal of publication:

Country:

Study design: measurement instrument:

Prospective cohort study
Case-control study
Cross-sectional study
Qualitative study
Other, namely:

Number of participants:
Characteristics of participant:

Primi/multipara:
Age:
Remarkable characteristics:

Outcome measures:

Time point of measurement postpartum:

Type of health problem discussed in this study:

Mental:
Social:
Physical:

Relevant results:

Strengths and limitations of the study

© 2023 Gmeling Meyling M. M. et al.
